# Evaluation of X-Inactivation Status and Cytogenetic Stability of Human Dermal Fibroblasts after Long-Term Culture

**DOI:** 10.1155/2010/289653

**Published:** 2010-12-27

**Authors:** Juan Chen, Zhan-Ping Shi, Juan Dong, Ting-Ting Liao, Yan-Peng Wang, Xue-Ping Sun, Zheng-Jie Yan, Xiao-Qiao Qian, Yu-Gui Cui, Zhi-Gang Xue, Guoping Fan, Jia-Yin Liu

**Affiliations:** ^1^Jiangsu Province Key Laboratory of Reproductive Medicine, Nanjing Medical University, Nanjing 210029, China; ^2^Center of Clinical Reproductive Medicine, The First Affiliated Hospital of Nanjing Medical University, Nanjing 210029, China; ^3^Stem Cell Research Center and Department of Regenerative Medicine, Medical School, Tongji University, Shanghai 200092, China; ^4^Department of Human Genetics, University of California Los Angeles, Los Angeles, CA 90095, USA

## Abstract

Human primary fibroblasts are a popular type of somatic cells for the production of induced pluripotent stem (iPS) cells. Here we characterized biological properties of primary fibroblasts in terms of cell-growth rate, cytogenetic stability, and the number of inactive X chromosomes during long-term passaging. We produced eight lines of female human dermal fibroblasts (HDFs) and found normal karyotype and expected pattern of X chromosome inactivation (XCI) at low passages (Passage P1-5). However, four out of the eight HDF lines at high passage numbers (≥ *P*10) exhibited duplicated hallmarks of inactive X chromosome including two punctuate signals of histone H3 lysine 27 trimethylation (H3K27me3) and X inactive-specific transcript (XIST) RNA signals in approximately 8.5–18.5% of the cells. Our data suggest that the copy number of inactive X chromosomes in a subset of female HDF is increased by a two-fold. Consistently, DNA fluorescent in situ hybridization (FISH) identified 3-4 copies of X chromosomes in one nucleus in this subset of cells with two inactive Xs. We conclude that female HDF cultures exhibit a higher risk of genetic anomalies such as carrying an increased number of X chromosomes including both active and inactive X chromosomes at a high passage (≥ *P*10).

## 1. Introduction

Fibroblasts are one of the most commonly used somatic cells to produce induced pluripotent stem (iPS) cells [1, 2]. Therefore, it is essential to determine the genetic and epigenetic stability of fibroblasts in long-term culture. It has been shown that long-term *in vitro* culture of somatic cells increases the risk of cell senescence, chromosomal aneuploidy, as well as multiploidy karyotypes [3, 4]. However, the potential changes in epigenetic gene regulation in long-term cultured human fibroblasts are not well investigated.

Dosage compensation of the X chromosome in mammalian female cells is one of classic examples of epigenetic gene regulation. It is achieved through an event referred to as X chromosome inactivation (XCI) in which one of the two X chromosomes is silenced during development [5–7]. XCI is initiated in the X-inactivation center of the X chromosome, within which a gene encoding X inactive-specific transcript (*XIST*) is expressed solely from the inactivated X chromosome. *XIST* gene is crucial for XCI because its noncoding RNA directly interacts with and coats the inactive X chromosome. After the completion of XCI, the two X chromosomes in female cells are distinguished by differential transcription of the *XIST* gene, DNA methylation, and histone modifications such as histone H3 lysine 27 trimethylation (H3K27me3) [8, 9].

In this study, human dermal fibroblasts (HDFs) were successfully generated from eight different female donors. We then examined the cell-growth rate, the stability of XCI in short- and long-term passages. We found that early passages of female HDFs retained a normal karyotype with the expected XCI pattern while high-passage cells exhibited a higher risk of carrying more than one copy of the inactive X chromosomes due to the presence of three or four copies of X-chromosomes in one nucleus. 

## 2. Material and Method

### 2.1. Derivation and Cell Culture of HDFs

This research project was approved and monitored by the Ethical Committee in the First Affiliated Hospital of Nanjing Medical University. Written informed consent was obtained from all 8 female patients before collecting their dermal tissue specimens. Tissue specimens were washed with phosphate buffered saline (PBS) solution with 100 U/mL penicillin and 100 mg/mL streptomycin. Fat and connective tissues were removed, and the epidermis was scraped off by an operating knife blade. The remnant tissues were sheared into clumps of approximately 1 mm^3^, which were then planted uniformly onto the bottom of 25 cm^2^ flasks. The flasks were then inverted and placed in a 5% CO_2_ incubator at 37°C. Eight hours later, the flasks were then turned over. Culture medium consists of Dulbecco's modified Eagle's medium (DMEM, pyruvate-free, high-glucose formulation; Invitrogen) supplemented with 20% fetal bovine serum (FBS; Invitrogen). Culture medium was changed every 3 days. After 7 days of incubation, we examined the HDF explants from tissue clumps. When the cells reach 80–90% confluence, the culture was split at a ratio of 1 : 2.

### 2.2. Monitoring Cell Proliferation Rate

Cells from passage 5 through 10 were cultured in a 6-well plate with a density of 4 × 10^4^ per well. The culture medium was again changed every 3 days. Six days later, the growth curves of 8 HDF cell lines were obtained by counting the number of cells per well every 24 hours.

### 2.3. Karyotype Analysis

Karyotype analysis on each HDF cell line was carried out via Giemsa-banding (G-banding) between passages 5–10. When 80–90% confluence was reached in the 25 cm^2^ flask, culture medium was changed and supplemented with 0.1 *μ*g/mL Colcemid solution (diluted from 10 *μ*g/mL stock, Cat. no. 15212-012. Invitrogen). After 15 hours, cells were collected and suspended in 5 mL of 0.075 M KCl solution. Then the suspension was incubated in 37°C for 30 minutes. 2 mL of fixation fluid (methanol: glacial acetic acid at 1 : 3) was added and mixed together, and the cells were then collected. After two rounds of fixations, the cell suspension was mounted onto slides and baked at 75°C for 3 hours. Routine chromosome G-banding analysis was then carried out. Twenty karyotypes per slide were examined.

### 2.4. Immunofluorescence and Fluorescence In Situ Hybridization (FISH)

#### 2.4.1. Cell Preparation

Cells at passage 1, 5, and 10 of each line were collected. Cells were plated on either slides or coverslips after being rinsed three times with 1x PBS for 5 minutes and then fixed with 4% paraformaldehyde for 20 minutes at room temperature. Then the cells were washed again with 1x PBS three times and stored in 70% EtOH at −20°C for subsequent analysis. All reagents used for RNA FISH were prepared with diethylpyrocarbonate- (DEPC-, Sigma) treated H_2_O. 

#### 2.4.2. XIST RNA FISH

 Cells were washed three times with 1x PBS and dehydrated with 70% ethanol for 2 minutes and 90% ethanol for 2 minutes. Then the cells were hybridized overnight at 37°C in hybridization buffer (50% formamide/2x standard saline citrate (SSC) using a Cy3-hXD1-3 probe as previously described in [10]. The next day, coverslips were rinsed twice with hybridization buffer for 30 minutes each at 37°C and then once with 1x PBS. Subsequently, after adding DAPI for 5 minutes, cells were mounted onto slides.

#### 2.4.3. H3K27me3 Immunofluorescence

The cells prepared as above were rinsed and incubated sequentially by the following procedures: cells were rinsed with 1x PBS three times, incubated in 1x PBS plus 0.4% Triton X-100 (Sigma) for 20 minutes and rinsed again with 1x PBS three times at room temperature. Blocking of nonspecific staining was conducted with 10% normalgoat serum in 1x PBS for 1 hour at room temperature, after which the cells were incubated overnight with a rabbit anti-H3K27me3 antibody (1 : 1000, from Update Biotechnology, USA) at 4°C in a humidified chamber. The cells were subsequently rinsed in 1x PBS three times and incubated for 1 hour in the dark with FITC-conjugated secondary antibody (1 : 200, BeiJing Zhong Shan Golden Bridge Biotechnology Co. Ltd, China). The cells were then rinsed with 1x PBS three times, counterstained with 4,6-diamidino-2-phenylindole (DAPI, Vysis, Inc., Downers Grove, IL, USA), and mounted onto slides after 5 minutes.

#### 2.4.4. X Chromosome DNA FISH

 Slides were immersed in 2x SSC solution jar, shook for 5 seconds, and removed after 10 minutes. Hybridization areas were made, and the temperature of the denaturing solution (70% formamide/2x SSC) was maintained at 73°C. After about 5 minutes, slides were dehydrated for 1 minute in 70% EtOH, followed by 1 minute in 85% EtOH, and then 1 minute in 100% EtOH. Concurrently, the probe mixture was prepared and placed in a 73°C water bath for 5 minutes. 10 *μ*L of probe mixture was then added to one target area after which the coverslip was immediately applied. Then the slides were placed in a prewarmed humidified box at 37°C overnight. The next day, the coverslip was removed from one slide and the slides were immediately immersed in the 0.4x SSC solution for 2 minutes at 70°C. Finally, the slides were air-dried in the dark and 10 *μ*L of counterstains were applied to the target area of the slides and the coverslips were added. A commercially available probe (Vysis, Inc., Downers Grove, IL, USA) targeting the centromeric region of X chromosome (green fluorescence) was used to evaluate the number of X chromosomes of the cells.

Signals were subsequently detected by a fluorescence microscope. Two hundred cells were examined randomly from each culture, and the numbers of signals in each cell nucleus were counted.

## 3. Results

### 3.1. Morphology and Proliferation Rate of HDF

**Figure 1:**
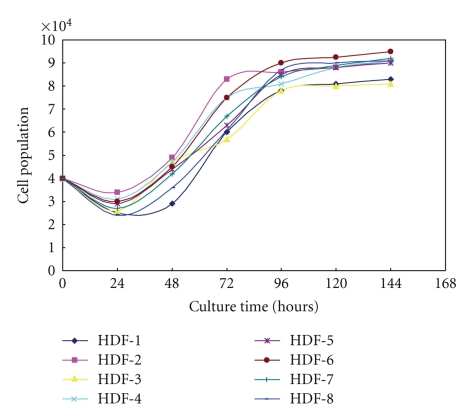
*Growth curves of 8 HDF lines.* The growth curves of eight cultured HDF cells show delitescence at 0–24 hours, exponential growth phase at 24–96 hours and stationary phase at 96–144 hours after cell passage.

HDFs were successfully isolated and expanded from 8 different female tissue specimens. After the first 24-hour inoculation, dermal tissues were completely adhered to the bottom of the flasks and HDFs began growing out in the following days. When HDFs reached 80–90% confluence, they were passaged as described in Materials and Methods. Within 10 passages, few of the cells exhibited senescence including intumescences, arborescence, and cell death. The growth curves showed that the number of HDF slowly increased in the first 24 hours after passage. From 24 to 96 hours, HDF proliferated exponentially. HDF cells entered stationary phase from 96 to 144 hours after passage (Figure 1). 

### 3.2. Karyotype Analysis of HDF

G-banding was conducted to determine the karyotypes of 8 HDF cell lines, and the results demonstrated that each culture exhibited normal karyotype in twenty metaphase spreads examined within the first 10 passages (data not shown). 

### 3.3. Analysis of X Chromosome Inactivation and Copy Number of X-Chromosome in Different Passages of HDFs

**Figure 2:**
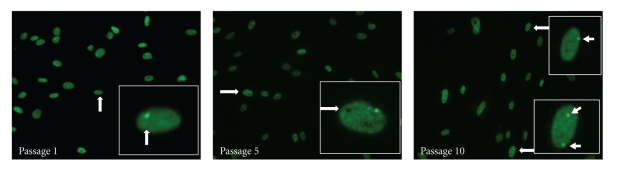
*Immunocytochemical (ICC) analysis of the number of inactive X chromosomes in HDF cultures.* HDFs in passages 1, 5, and 10 showed one positively stained H3K27me3 punctate signal (arrow) in most nuclei. Two green signals (arrows), inactive of two inactive Xs, were observed in few of HDF cells at passage 10.

**Figure 3:**
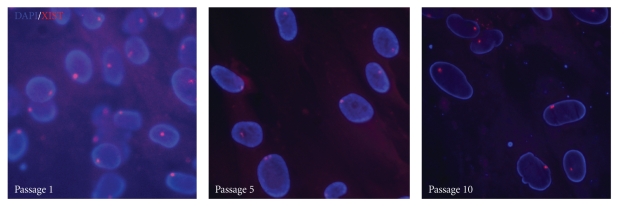
*XIST RNA FISH analysis of the number of inactive X chromosomes.* FISH analysis of HDF cultures in passages 1, 5, and 10 showed that most of the nuclei counterstained with DAPI (in blue) have one small spot signal for *XIST* RNA (in red) in the merged image. This was in accordance with the results of the H3K27me3 ICC analysis in Figure 2.

 H3K27me3 immunostaining and *XIST* RNA FISH were employed to detect XCI patterns. One intense punctate signal of H3K27me3 was found in most HDF nuclei within passages 1 and 5 (Figure 2). Occasionally, we also found cells that exhibit either zero or two punctuate staining signals. After randomly selecting and counting the number of nuclei with 0, 1, and 2 H3K27me3 signals, we found that the number of cells without H3K27me3 punctate signals appeared to increase at a higher passage number (Table 1). The *XIST* RNA FISH analysis yielded the same results as H3K27me3 immunostaining (Figure 3).

**Figure 4:**
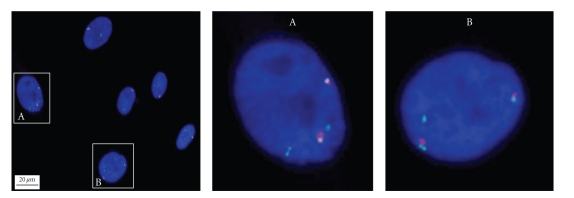
*FISH analysis of the number of total X-chromosomes as well as the number of inactive Xs.* The total number of X-chromosomes in HDF cells at passage 10 was detected by DNA FISH analysis of the centromeric region of X chromosome (green) and the number of inactive X chromosomes by *XIST* RNA FISH assay (red). Note that the double signals for *XIST *RNA are indicative of the two inactive Xs, which can be observed in cell nucleus with either quadruple-signals for X chromosome (A) or triple signals for X chromosome (B). Cell nucleus was counterstained with DAPI (blue).

Interestingly, in four out of total eight cell lines (#1, 2, 3, and 8), our counting data showed that two positive signals of *XIST* RNA coating or H3K27me3 staining of inactive X chromosomes were present in approximately 8.5 ~ 18.5% HDF cells at passage 10 (Figures 2 and 4). Consistent with the increased number of inactive X-chromosome, a few cells showed three or four copies of X chromosome signals from DNA FISH analysis of total number of X chromosomes. Our counting data showed that approximately 6% of the cells exhibited three copies of X chromosomes and 16% with four copies of X-chromosomes (Figure 4).

## 4. Discussion

Pluripotent cells are considered a major resource for regenerative medicine, and fibroblasts are the most commonly used cell type to produce iPS cells by reprogramming [1, 2]. In order to produce quality iPS cells, the biological properties of fibroblasts must first be characterized prior to reprogramming. These include cell growth state, stability of cytogenetic and epigenetic status such as X chromosome inactivation.

As a pioneer on cell senescence study, Hayflick was the first to establish a reliable protocol to maintain fibroblast growth *in vitro* [11]. Early studies showed that HDFs can reach over 50 population doublings before senescence sets in, but its proliferative capacity depends on the age of the donor and the biopsy site [12]. In our cultures of eight female HDF lines, cells with fusiform shape and senescence phenotype are minimal in early passages, indicating that cells within passage 10 are healthy. Nevertheless, *in vitro* cultures may still affect genetic and epigenetic stability of the cultured HDFs. By counting the cell numbers after passaging, we plotted the cell growth curves which demonstrated that cells were in delitescence in the first 24 hours after cell passage. The cells then proliferated rapidly in exponential growth phase from 24 to 96 hours and entered stationary phase from 96 to 144 hours (Figure 1).

Previous *in vitro* culture studies report spontaneously mutated clones in most species, especially in rodents, usually after 5–7 passages. These mutated clones have abnormal karyotypes including chromosomal aneuploidy and multi-ploidy karyotypes [3]. In our results, we did not find any definitively abnormal karyotypes through the standard G-banding karyotyping. However, in a detailed analysis of X-inactivation status, we found that a portion of cells (8.5–18.5%) in half of our cultures (four out of eight HDF lines) exhibited more than two copies of X-chromosomes. Our data suggest that identification of the number of inactive X chromosomes by XIST RNA FISH or the total number of X chromosomes by DNA FISH analysis is a more sensitive method to detect chromosomal variations than the standard G-banding karyotyping.

XCI is a fundamental epigenetic mechanism in female somatic cells, involving the dosage compensation of X-linkage genes [13]. Any improper changes in the dosage of X-linkage gene would impair the normal function of somatic cells. Research on human female embryonic stem cell (ESC) lines indicated that XCI patterns of these lines could change during passaging. *XIST* gene expression in cultured hESC lines was unstable and subjected to epigenetic silencing by DNA methylation [10]. In this study, *XIST* gene RNA FISH and H3K27me3 immunofluorescence were performed to detect the XCI status in passages 1, 5, and 10 of HDF cells. The results indicated that in most cell nucleus only one XCI signal (green spot for H3K27me3 or red spot for *XIST* RNA) was observed, and few of the cells had no XCI markers (Figures 2 and 3). Cell numbers without XCI signals tended to increase along with HDF population doublings (Table), which could be DNA methylation on the *XIST* promoter to silence its expression [10]. Additionally, two *XIST* RNA signals in coupling with 3-4 signals of X chromosome can be observed in the same nucleus in a few cells at passage 10 (Figure 4). The presence of two *XIST* RNA signals indicate two copies of inactive X chromosomes, consistent with the increased number of total X-chromosomes.

In summary, our present study demonstrates that early passages of female HDF cultures are genetically more stable than cultures after 10 passages. By monitoring XCI status and the number of inactive X chromosome, we can effectively determine whether the chromosome ploidy of human female somatic cells changes over the course of cell culture. The information of genome stability is important for evaluating the suitability of these cells for other applications such as the derivation of iPS cells in regenerative medicine.

## Figures and Tables

**Table 1:** Quantitation of the percentage of HDF cells without, or with one or two punctate signal(s) for H3K27me3 staining in HDF no.1–8.

Cell line	Passage 1			Passage 5			Passage 10		
	S0	S1	S2	S0	S1	S2	S0	S1	S2
HDF-1	34	166	0	44	156	0	47	137	26
HDF-2	28	172	0	38	162	0	37	131	32
HDF-3	40	160	0	48	152	0	52	111	37
HDF-4	26	174	0	31	169	0	80	120	0
HDF-5	22	178	0	32	168	0	62	138	0
HDF-6	42	158	0	44	156	0	64	136	0
HDF-7	39	161	0	51	149	0	74	126	0
HDF-8	24	176	0	41	159	0	67	116	17

S0, S1, and S2, respectively, represent zero, one, and two H3K27me3 signal(s) observed in each nucleus.
